# Supplementary material for: Formation of Nanotwin Networks during High-Temperature Crystallization of Amorphous Germanium
Source: Sci Rep. 2015 Nov 26;5:17251. doi: 10.1038/srep17251 (PMC4660462; doi:10.1038/srep17251)
Supplement: Supplementary Information [file srep17251-s1.pdf]

Formation of Nanotwin Networks during High-Temperature Crystallization of Amorphous Germanium

Luis Sandoval[1], Celia Reina[2], Jaime Marian[3]

[1]Los Alamos National Laboratory, Los Alamos, NM 87545, United States
[2]University of Pennsylvania, Philadelphia, PA 19104, United States
[3]University of California Los Angeles, Los Angeles, CA 90095, United States

Supplementary Information – Video legends:

Video 1
Legend: Animation of the MD simulation showing full atomistic details of the grain growth process in Ge at 1100 K. This video accompanies Figure 5(a). Only atoms with a crystalline structure are shown (the embedding amorphous matrix is not shown for clarity). To get a view of the internal twin boundaries, only a slice of the total grain is displayed, which is rotated for better visualization. Dark blue and orange spheres represent atoms with diamond cubic and twin plate structure, respectively. Light blue spheres represent atoms belonging to the amorphous/crystalline interface and dislocation cores.

Video 2
Legend: Animation of the MD simulation showing the creation and evolution of interfaces during the grain growth process in Ge at 1100 K. This video accompanies Figure 5(b). The video shows the growth of the external a/c interface, as well as internal twin boundaries bounding crystalline subgrains. The system is rotated during the animation for better visualization.

Video 3
Legend: Animation of the MD simulation of an [111]-oriented a/c bi-crystal showing the spontaneous crystallization of the entire box at 1100 K. Only atoms with a crystalline structure are shown. Profuse twin formation is observed. Dark blue and orange spheres represent atoms with diamond cubic and twin plate structure, respectively. Light blue spheres represent atoms belonging to the amorphous/crystalline interface.

Video 4
Legend: Animation of the MD simulation of an [011]-oriented a/c bi-crystal showing the spontaneous crystallization of the entire box at 1100 K. Only atoms with a crystalline structure are shown. No twinning is observed. Dark blue and orange spheres represent atoms with diamond cubic and twin plate structure, respectively. Light blue spheres

represent atoms belonging to the amorphous/crystalline interface.

Video 5
Legend: Animation of the MD simulation of an [001]-oriented a/c bi-crystal showing the spontaneous crystallization of the entire box at 1100 K. Only atoms with a crystalline structure are shown. No twinning is observed. Dark blue and orange spheres represent atoms with diamond cubic and twin plate structure, respectively. Light blue spheres represent atoms belonging to the amorphous/crystalline interface.
